# Nano-fluorescence imaging: advancing lymphatic disease diagnosis and monitoring

**DOI:** 10.1186/s40580-024-00462-1

**Published:** 2024-12-11

**Authors:** Chae Yeon Han, Sang-Hun Choi, Soo-Hyang Chi, Ji Hyun Hong, Young-Eun Cho, Jihoon Kim

**Affiliations:** 1 School of Integrative Engineering, Chung-Ang University, Seoul, 06974 South Korea; 2 Department of Radiation Oncology, Seoul St. Mary’s Hospital, College of Medicine, The Catholic University of Korea, Seoul, 06591 South Korea; 3 Department of Food and Nutrition, Andong National University, Andong, 36729 South Korea

**Keywords:** Nano-fluorescence, Lymph node, Fluorescence imaging

## Abstract

The lymphatic system plays a crucial role in maintaining physiological homeostasis and regulating immune responses. Traditional imaging modalities such as magnetic resonance imaging, computerized tomography, and positron emission tomography have been widely used to diagnose disorders in the lymphatic system, including lymphedema, lymphangioma, lymphatic metastasis, and Castleman disease. Nano-fluorescence technology has distinct advantages—including naked-eye visibility, operational simplicity, portability of the laser, and real-time visibility—and serves as an innovative alternative to traditional imaging techniques. This review explores recent advancements in nano-fluorescence imaging aimed at enhancing the resolution of lymphatic structure, function, and immunity. After delineating the fundamental characteristics of lymphatic systems, it elaborates on the development of various nano-fluorescence systems (including nanoparticles incorporating fluorescent dyes and those with intrinsic fluorescence) while addressing key challenges such as photobleaching, limited tissue penetration, biocompatibility, and signal interference from biomolecules. Furthermore, this review highlights the clinical applications of nano-fluorescence and its potential integration into standard diagnostic protocols. Ongoing advancements in nanoparticle technology underscore the potential of nano-fluorescence to revolutionize the diagnosis and treatment of lymphatic disease.

**Graphical abstract:**

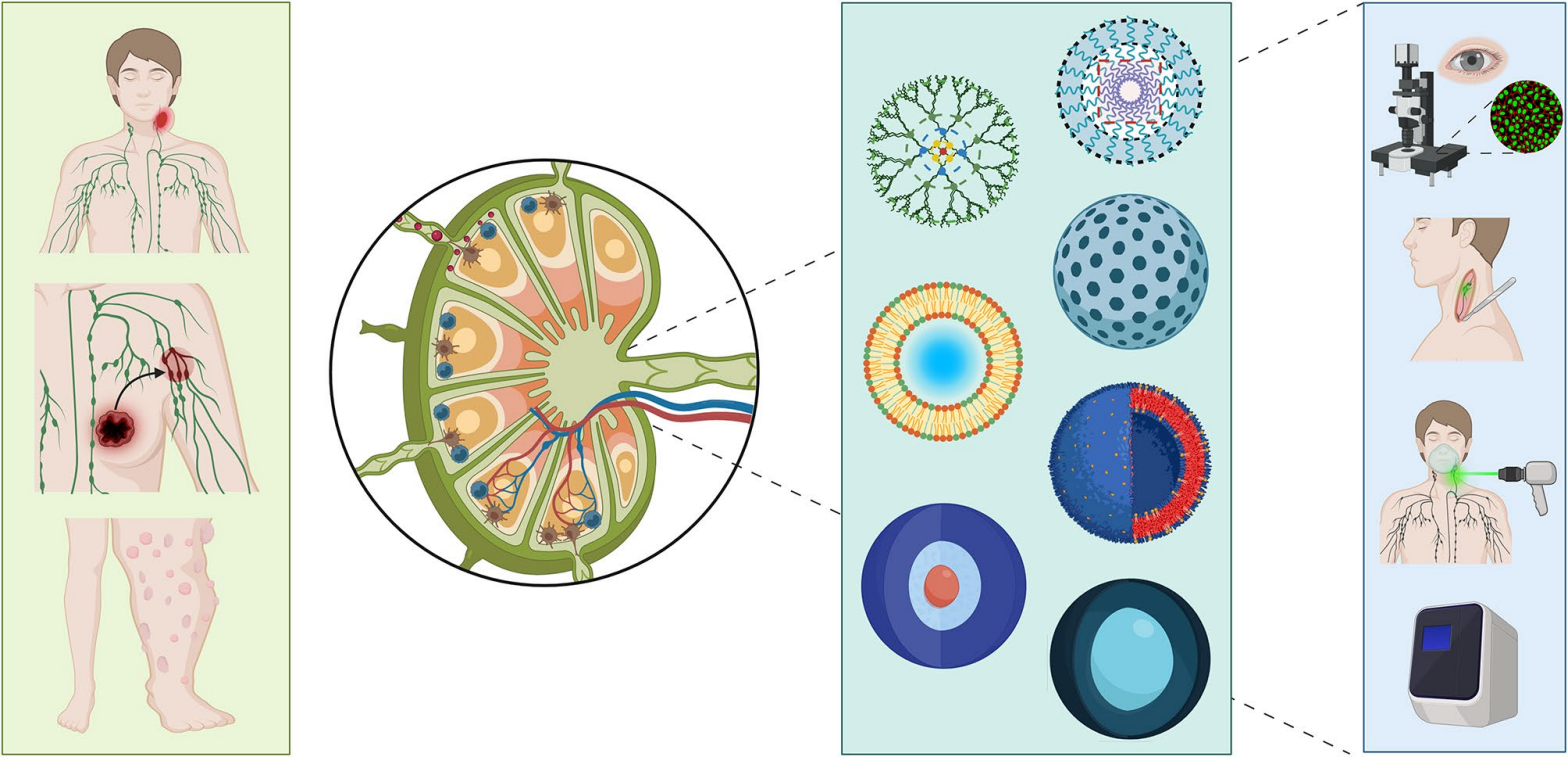

**Supplementary Information:**

The online version contains supplementary material available at 10.1186/s40580-024-00462-1.

## Introduction

**Fig. 1:**
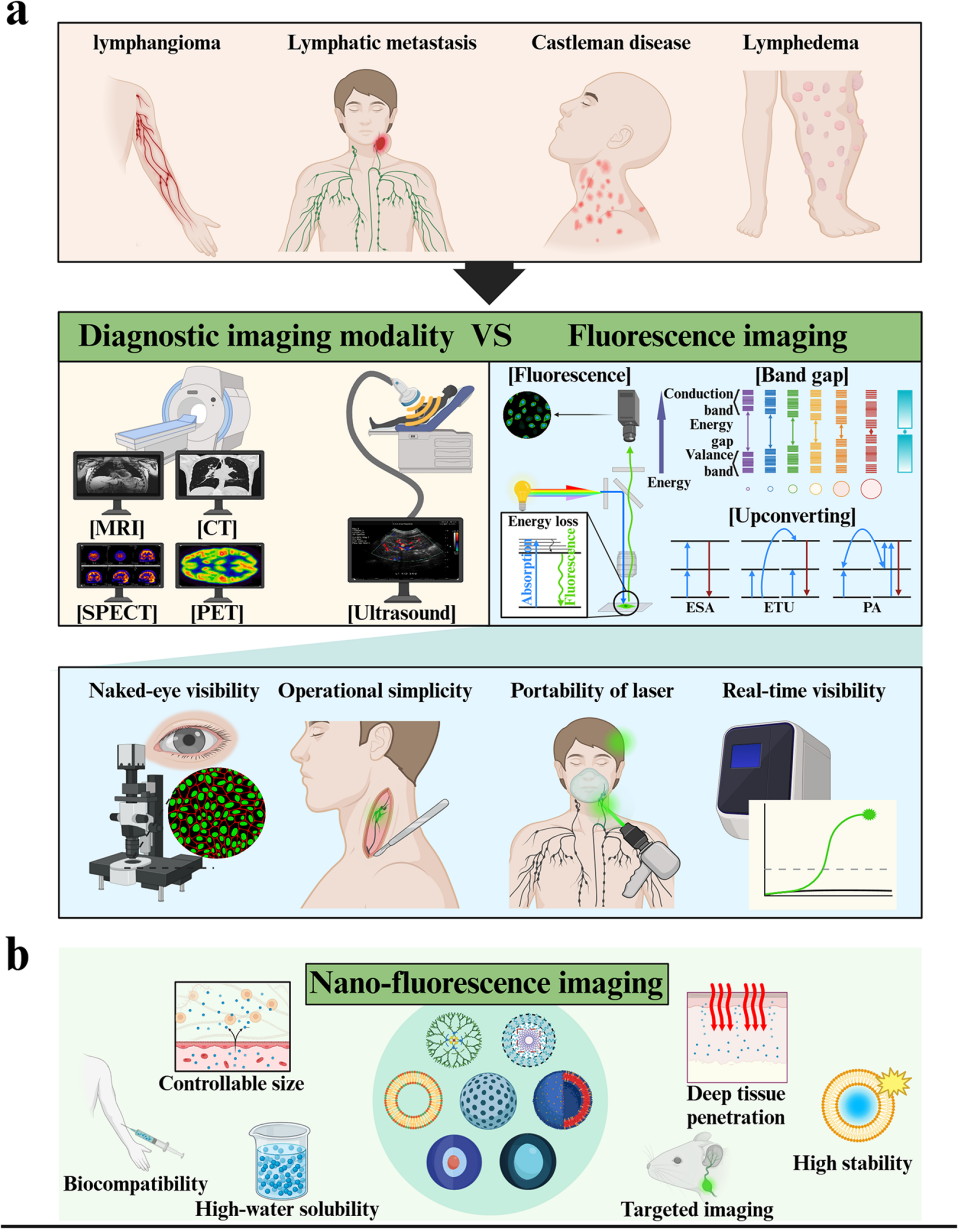
Schematic advantages of nano-fluorescence imaging. (**a**) Advantages of fluorescence imaging over various diagnostic imaging modalities in the diagnosis of lymphatic disease (**b**) Features of various nanoparticles in fluorescence imaging

Lymph nodes (LNs) and lymphatic vessels (LVs) not only manage homeostasis throughout the body by collecting excess fluid from peripheral tissues and returning it to the blood circulation system, but also by filtering pathogens and harmful substances and providing immunity. Disorders of LNs and LVs including lymphangitis, lymphangioma, lymphocytosis, lymphatic filariasis, Castleman disease, lymphangioleiomyomatosis, lymphedema, and mesenteric lymphadenitis have also been observed (Fig. 1) [1–5]. In addition, cancer metastasis (i.e., the spread of cancer cells from their original locations to other parts of the body) is accompanied by the initial invasion of cancer cells into tumor-draining LNs (TdLNs) through LVs. The clinical relevance of LNs’ physiology and immunity in various diseases has been recognized and emphasized in many studies [6–8]. Decades of content have demonstrated that imaging LNs plays a crucial role in the diagnosis and prognosis of cancer patients [9–11]. The abnormal size and morphology of sentinel LNs (SLNs), which drain primary tumors, may indicate metastasis and active immune response (Fig. 1), whereas activated T cells in patients’ LNs correlate with improved survival outcomes. In patients with lymphedema, early detection facilitates a prompt intervention to prevent disease progression [12–14]. Thus, lymphatic diagnosis remains a major challenge in predicting the prognosis of patients with diverse lymphatic-associated diseases and providing a proper treatment regimen.

In clinical practice, numerous diagnostic imaging modalities—including ultrasound (US), magnetic resonance imaging (MRI), computed tomography (CT), single photon emission CT (SPECT) and positron emission tomography (PET)—are commonly employed to diagnose the physiology of LNs and LVs (Fig. 1) [10, 15–24]. US is a fast, non-invasive, and inexpensive diagnostic tool to provide images in real-time despite its low resolution, limited detection when the target tissues contain gas or are located behind hard tissues (e.g., bones), and high dependency of diagnostic results on a skilled operator [25, 26]. MRI is a non-ionizing, non-radioactive technique that produces high-spatial-resolution images with unlimited tissue penetration; however, it is time-consuming and has low sensitivity [27]. Despite the radiation risk, CT facilitates the acquisition of 3D, high-spatial-resolution images with unlimited tissue penetration. SPECT and PET have been lauded for their high sensitivity and quantitative imaging data with unlimited tissue penetration. However, they have been criticized for their radiation risk and high cost owing to the use of radionuclides [28]. Various imaging agents have also been developed and exploited to enhance diagnostic accuracy.

Fluorescence imaging modalities are grounded in the use of fluorescent substances that are excited by light sources and emit light at wavelengths longer than the excitation wavelength. This technique is a promising approach owing to its distinct advantages over other modalities, including visibility to the naked eye, operational simplicity, laser portability, and real-time visibility (Fig. 1) [10, 29, 30].

Notwithstanding, several significant concerns must be addressed before practical use. The primary problem is the poor quantum yield of conventional fluorescent dyes [31]. One alternative for improving fluorescence is quantum dots (QDs), which were highlighted in the context of the Nobel Prize in Chemistry [32]. The secondary issue is the low signal-to-noise ratio owing to signal interference by biomolecules and the limited tissue penetration of ultraviolet (UV)/visible light in terms of the excitation of fluorescent substances and the detection of emitted light [31, 33]. Various biomolecules naturally present in biological tissues—including red blood cells, collagen, elastin, proteins, and even water—exhibit autofluorescence, making it difficult to accurately measure the desired signal [34–38]. In addition, organic fluorophores face inherent limitations in generating meaningful fluorescent signals at depths greater than 1–2 cm due to insufficient light absorption and emission properties in vivo [31]. To address these concerns, near-infrared (NIR) light and NIR-responsive fluorescent substances including NIR nano-fluorescent probes, QDs, and upconversion nanoparticles (UCNPs) have been investigated [39]. The third challenge is the difficulty in visualizing specific tissues, cells, and biomolecules of interest. An important technique for achieving targeted imaging includes nanoformulations that exploit the enhanced permeability and retention (EPR) effects of the tumor microenvironment and ligand modifications that bind to biomolecules specifically expressed or overexpressed in the cells of interest [18, 27, 40–46]. Finally, fluorescent probes must be highly biocompatible to avoid severe adverse reactions within the body [47].

Nanoformulations with carefully optimized physicochemical properties (e.g., size, surface charge, surface moiety) have a significant impact on enhancing the performance of fluorescent probes for in vivo imaging, enabling them to circulate sufficiently within the body, reach the desired locations, demonstrate enhanced in vivo performance, and be safely expelled after fulfilling their purpose [48–50]. These physicochemical properties also significantly govern NP transport to LNs through interstitial spaces. In addition, nanoformulations not only can help to solve the issue of biomolecule-mediated photobleaching, but also can prevent fluorescent substances from interacting directly with biomolecules and phagocytosis by various cells [50–56]. In short, nano-fluorescence in the form of nanoformulations of fluorescent dyes and NPs emitting fluorescence on their own can be an alternative to lymphatic fluorescence imaging while addressing the concerns associated with fluorescence imaging.

Several Food and Drug Administration (FDA)-approved fluorescence dyes—including indocyanine green (ICG), methylene blue, 5-aminolevulinic acid (5-ALA), hexaminolevulinate, and pafolacianine—are currently available as fluorescence imaging agents [57]. As a representative FDA-approved cyanine derivative NIR fluorescent dye, ICG-mediated lymphography has been utilized to examine subcutaneous lymphatic functions in clinics [58–62]. IRDye 800CW is another cyanine derivative NIR dye, which also widely used in clinical settings and currently being tested in multiple clinical trials, including head and neck cancer, breast cancer, pancreatic cancer, and brain tumor [59, 63, 64]. Methylene blue, 5-ALA, ICG and fluorescein sodium are approved by the FDA for image-guided surgery [65–68]. In addition, fluorescein sodium has been used in the diagnosis of retinal and iris vascular conditions, as well as cerebral aneurysm, through angiography [59, 64]. In particular, methylene blue is not only commonly utilized as a contrast agent for imaging the gastrointestinal tract and methemoglobinemia, but also is exploited in SLN mapping for breast cancer, along with ICG, in clinical settings [59, 69]. Furthermore, numerous NP-based imaging agents have been explored in clinical trials. These efforts are expected to pave the way for new opportunities for nano-fluorescence in clinical settings for the diagnosis and treatment of lymphatic-associated illnesses. This review addresses current nano-fluorescence technology used to detect LN physiology and immunity. First, we outline the lymphatic system’s vital functions and structures. Next, we discuss the content development and use of nano-fluorescence to identify the locations of LNs, physiological changes, and immune cell trafficking.

## Lymphatic function and structure

**Fig. 2:**
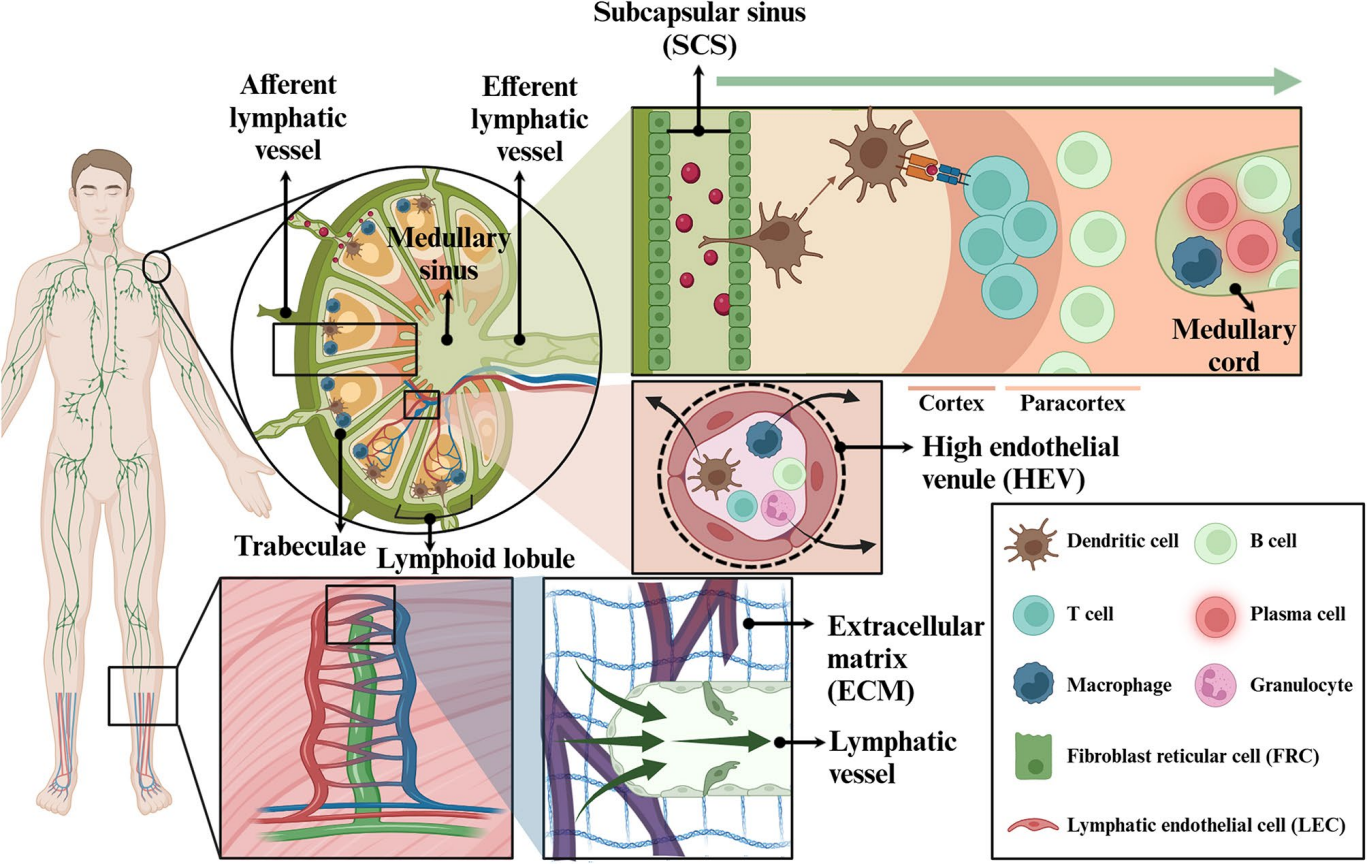
Graphical illustrations of lymphatic transporting and immune systems

Fluids, cells, and solutes move from the peripheral tissues to the LNs, accompanied by the initial blind-ended lymphatic capillaries. Lymphatic capillaries are lined with overlapping lymphatic endothelial cells (LECs) that feature incomplete intercellular junctions, allowing interstitial fluid to pass through the extracellular matrix (ECM) and enter the capillaries more easily [53, 70–74]. The lymph, defined as the interstitial fluid entering the lymphatic system, is then transported from the initial lymphatic capillaries to larger LVs. As the lymph moves through these vessels, it is directed by a system of one-way valves that ensure it flows in a single direction towards a collecting duct, a larger tube within the lymphatic system [75, 76]. The lymph enters the LN through afferent LVs and first reaches the subcapsular sinus (SCS) [72, 77]; it then progresses through the outer cortex of the LN and continues through the inner medulla. LN-borne immune cells are responsible for sampling and filtering the lymph to identify and process pathogens and harmful substances. The now-filtered lymph is drained from the LNs through the efferent LVs, progressively moving into the larger LVs. Finally, the lymph returns to the bloodstream via the thoracic or right lymphatic duct, which drains into the subclavian veins. High endothelial venules (HEVs), which are encased by fibroblast reticular cells (FRCs), are located in the post-capillary venular network that connects the LN-incoming arteries in the outer cortex or B-cell zone and the medullar collecting veins in the medulla [78, 79]. HEVs provide another pathway for lymphocytes—including T cells, B cells, and natural killer (NK) cells—to enter LNs [80, 81]. In short, the lymphatic system plays a crucial role in maintaining fluid balance by transporting interstitial fluid leaked from blood vessels to the LNs and returning it to the bloodstream; it also plays a significant role in immunosurveillance and immune cell movement.

An LN has a small bean-shaped structure supported by a reticular meshwork composed of delicate FRCs and reticular fibers (Fig. 2). This structure aids in the movement and distribution of lymphocytes within the LN. The outermost layer of an LN appears capsule-shaped with fibrous connective tissue encasing the entire node, protecting the LN and providing structural support [82]. Extensions of the capsule, called trabeculae, penetrate the node, divide it into compartments, and provide pathways for blood vessels [83]. Beneath the capsule lies the cortex, which is further divided into two regions: the outer cortex and inner cortex (paracortex) [72, 83]. The outer cortex primarily contains B-cells organized in structures called follicles, which are spherical clusters of lymphocytes. Immature B lymphocytes are activated in response to antigens and proliferate and produce antibodies. The paracortex, where antigen presentation and T lymphocyte activation occur, is mostly populated by T lymphocytes and dendritic cells (DCs). Between the capsule and cortex lies the SCS, the first space into which lymph flows upon entering the LN. Below the cortex, the medulla is composed of medullary cords and sinuses. Medullary cords are densely packed with B cells, macrophages, and plasma cells, whereas medullary sinuses are spaces through which lymph fluid flows, allowing it to exit the LN.

Interstitial fluid is essential for the transport of materials from the peripheral tissues and site of locoregional injection to the LN since diffusion, an effective transport mechanism, becomes less efficient for longer distances or larger particles. Nano-sized materials typically hitchhike interstitial fluid within the tissue, are absorbed into the initial lymphatics, and thereby drain into the LNs. There are several factors that significantly influence the lymphatic drainage, including surface charge, rigidity, and particularly size [53]. Small particles (< 10 nm) tend to rapidly diffuse into blood vessels following subcutaneous injection and contribute to low accumulation in the LNs [84]. NPs in the range of 10–100 nm can leverage interstitial flow, enter the lymphatic vessels, and subsequently accumulate in the LNs [53, 72, 84–86]. The lymphatic drainages are also influenced by the rigidity of the materials involved. Despite their appropriate size for LN penetration, rigid nanoparticles struggle more to efficiently pass through the pores of the ECM because of their stiffness compared to flexible ones [87–89]. Additionally, positively charged substances face electrostatic interactions, limiting their ability to efficiently be transported to the LN because the cellular membranes in ECM exhibit a negative charge [90]. Instead, positively charged nanoparticles have shown higher cellular uptake by immune cells, which allows phagocytic immune cells-mediated lymphatic transport [91]. Nevertheless, strong positive charges on the nanoparticles can induce toxicity by damaging cell membranes and aggregation with various negatively charged proteins in the body, which makes them less suitable for LN delivery [92]. Accordingly, comparative studies have shown that nanoparticles with weak negative charges are more effectively delivered to the LNs than those with neutral or positive charges [93, 94].

Therefore, the intricate structures, physiology, and functions of lymphatic systems in relation to the physicochemical properties of materials that influence lymphatic transport, must be thoroughly considered to achieve precise detection and diagnosis of LNs, LVs, and immunity – key determinants of the prognosis and treatment of lymphatic-associated diseases. In the following sections, we review several nano-fluorescence technologies that enable the detection and visualization of LNs, LVs, and lymphocytes, leveraging nanoformulations and fluorescence imaging techniques. Their clinical significance in lymphatic-associated diseases is also discussed.

## Lymphatic fluorescence imaging using NPs

**Fig. 3:**
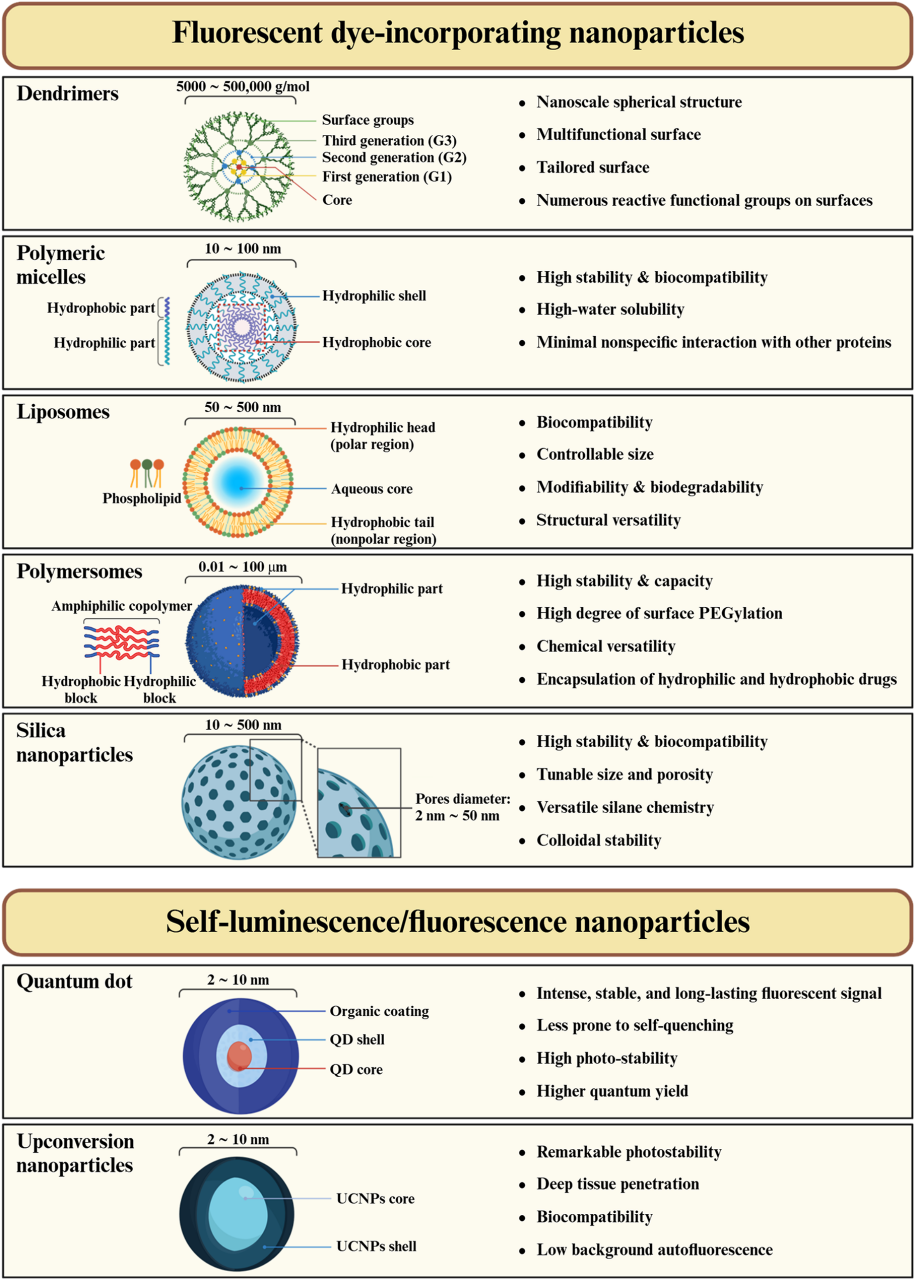
Various types of NPs in lymphatic fluorescence imaging and their categorization into fluorescent dye-incorporating NPs and self-luminescence/fluorescence NPs

Nano-fluorescence for lymphatic imaging can be categorized into two main strategies (Fig. 3). The first one involves incorporating fluorescent dyes (e.g., polymeric micelles, liposomes, polymersomes, silica NPs) into NPs that do not emit fluorescence on their own [44]. The second strategy entails the use of NPs that intrinsically exhibit fluorescence (including QDs and UCNPs). Accordingly, here we address the development and use of various nano-fluorescence techniques to examine the location, distribution, and physiology of LNs.

### Fluorescent dye-incorporating NPs

Nano-fluorescence can be easily achieved by physically loading or chemically conjugating fluorescent dyes onto NPs that do not inherently exhibit fluorescence. Among numerous fluorescent dyes, those that emit NIR light, typically ranging from 700 to 1700 nm, are preferred due to their superior tissue penetration and reduced signal interference with in vivo biomolecules [57, 95]. Representative NIR dyes include cyanine-based dyes, such as Cy5, Cy5.5, Cy7, and ICG. Herein, we introduce the development of fluorescent dye-incorporated nano-fluorescence and explore its potential in the diagnosis of lymphatic-associated diseases.

#### Dendrimers

Dendrimers are branched macromolecules with sizes ranging from 5,000 to 500,000 g/mol. Unlike hyperbranched polymers, dendrimers feature a well-defined nanoscale spherical structure with a multifunctional surface, enabling tailored surface chemistry and potential for drug and imaging agent entrapment (Table 1). Polyamidoamine (PAMAM) dendrimers synthesized using divergent or convergent methods have been extensively explored for biomedical applications [96]. Their suitability for creating macromolecular imaging contrast agents is underscored by several key features, including well-defined structures, distinct sizes, and numerous reactive functional groups on their surfaces. PAMAM dendrimers are nanoscale structure with negative surface charges and terminal functional groups, which enable them to pass through the ECM and accumulate in the lymphatic system as described in Fig. 3 [53, 97]. For instance, PAMAM dendrimers have been functionalized with Gd-(III)-DTPA chelates and the NIR fluorescent dye Cy5.5, establishing them as dendrimer-based dual MRI and fluorescence imaging agents [98]. Fluorescence imaging effectively detected LNs with high sensitivity despite the use of a relatively small amount of Cy 5.5 dye compared to Gd(III) ions. While a 25 µL solution of 30 mM Gd was required for consistent LN identification with a 3-T MRI scanner, fluorescence imaging could detect the nodes with as little as 0.5 nmol of Cy 5.5. However, fluorescence imaging showed some blurring of the LN edges due to light diffusion, whereas MRI provided a clearer view of both the LN and surrounding LVs. This work suggests that MRI can be used to map SLNs prior to surgery, and that fluorescence imaging can guide surgeons to precisely locate LNs during the procedure.

#### Polymeric micelles

Polymeric micelles are a promising platform for enhanced drug delivery and are formed by the self-assembly of amphiphilic polymers into a spherical core-shell structure in aqueous solutions [99–102]. Amphiphilic polymers consist of two distinct parts: a hydrophobic core and a hydrophilic shell. The hydrophobic core is primarily used as a drug reservoir, whereas the hydrophilic shell protects the drug-loaded core. The size of polymeric micelles typically ranges from 10 to 100 nm, which is ideal for passive transport and allows for efficient delivery to LVs through interstitial flow [103]. Accordingly, strategies utilizing polymeric micelles for LN targeting have been studied extensively (Table 1).

1,2-distearoyl-*sn*-glycero-3-phosphoethanolamine-poly(ethylene glycol) (DSPE-PEG) is an FDA-approved polymer with high stability and biocompatibility, as shown in Fig. 3 [104–109]. DSPE-PEG can self-assemble into polymeric micelles with a size appropriate for lymphatic uptake; doxorubicin loaded DSPE-PEG micelle (MPEG-DSPE/DOX) exhibits a size of 20 ± 5 nm [110]. An additional load of lipophilic NIR-emitting 1,1’-dioctadecyl-3,3,3’,3’-tetramethyl indotricarbocyanine iodide (DiR) fluorescent dye, with an excitation at 754 nm and emission at 778 nm, facilitated in vivo fluorescence imaging to assess the delivery efficiency of MPEG-DSPE micelles to the LNs. After subcutaneous injection of MPEG-DSPE/DiR micelles into the left footpad of nude mice, the accumulation of micelles in the left popliteal LN (PLN) significantly increased over time compared to the right side. These results suggest that the MPEG-DSPE micelles were taken up by the lymphatic drainage system and accumulated in the LNs. PEG-DSPE micelles were further modified with mannose, a ligand for CD206 overexpressed on tumor-associated macrophages (TAMs) enriched in tumor microenvironments, enabling the diagnosis of LN micrometastasis [111]. Hydrophilic mannose was conjugated with hydrophobic IR780 via disulfide bonds to afford the mannose-IR780 conjugate (MR780), which can be co-embedded into PEG-DSPE micelles with a size of 72.81 ± 4.39 nm. The NPs could accumulate and then exhibit higher fluorescence intensity in metastatic LNs than in normal LNs not only due to the selective interactions between mannose and TAMs but also the recovery of fluorescence signals by the cleavage of disulfide bonds between mannose and IR780 in tumor microenvironments with high redox conditions.

**Fig. 4:**
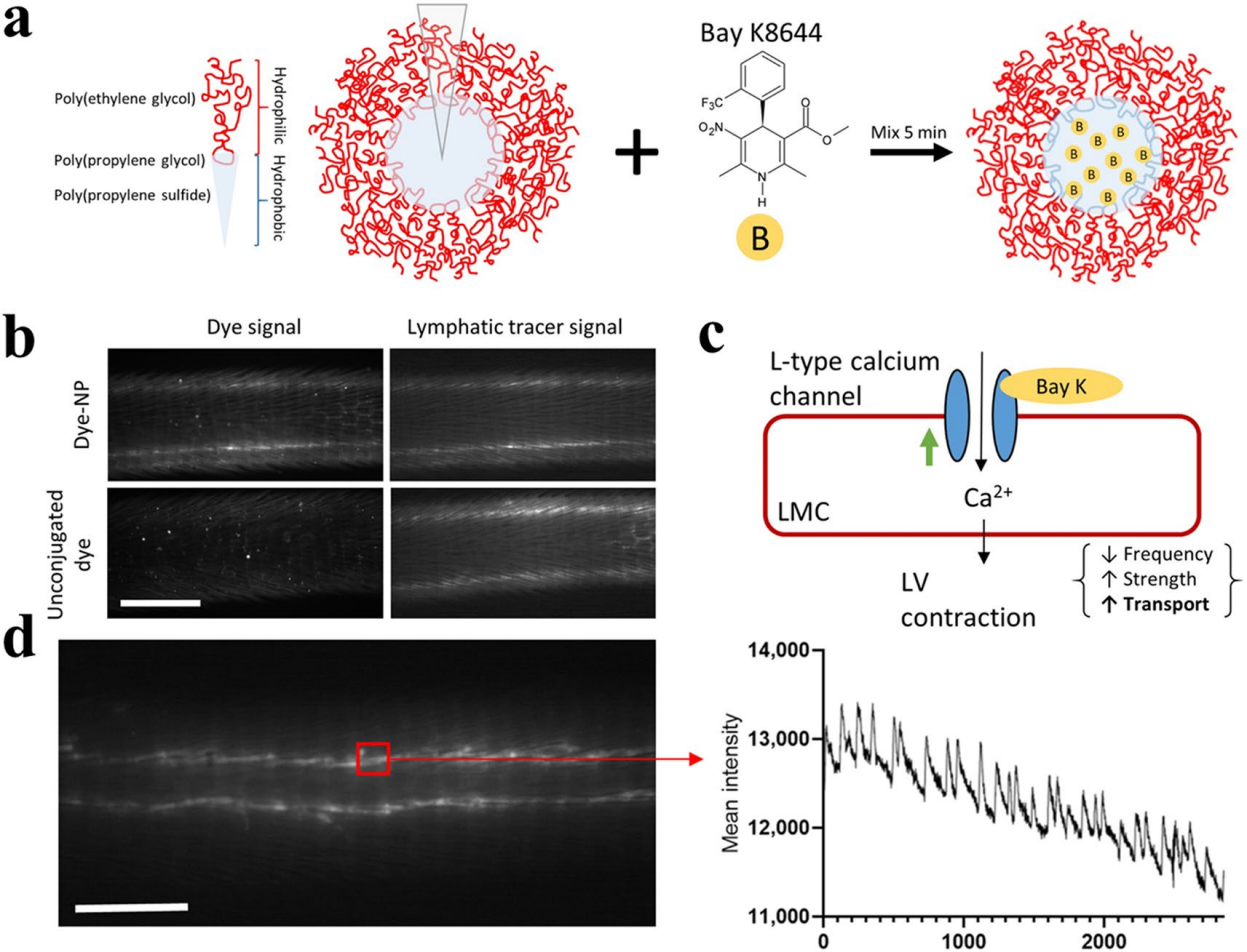
Fluorescence dye-loaded micelles in the investigation of lymphatic pumping in vivo. (**a**) A schematic structure of PPS/PPG-PEG NPs incorporating the hydrophobic molecule BayK. (**b**) Visualization of free dye and dye-loaded micelles in the tail collecting LVs (left) following co-injection with a lymphatic-draining PEG tracer (right). Scale bar, 3 mm. (**c**) A schematic mechanism to depict the impact of BayK on LVs’ pumping function. (**d**) NIR image of LVs in the tail, with corresponding fluorescent intensity traces analyzed to derive pumping metrics for marked regions of interest. Scale bar, 2 mm. (This figure is reproduced from [114], an open-access article distributed under the terms of the Creative Commons Attribution (CC BY-NC) license.)

Thomas et al. utilized NIR dye-labeled polymeric micelles consisting of a hydrophobic core made of poly(propylene sulfide) (PPS) and poly(propylene glycol) (PPG), along with a hydrophilic shell of PEG, to explore the dysfunction of LVs pumping in lymphedema (Fig. 4a) [112–120]. The IRDye680-labeling PPS/PPG-PEG micelles with a diameter of 30 nm could clearly visualize the LVs, whereas the fluorescence signals of free IRDye680 were not observed in the LVs (Fig. 4b) [114]. Changes in LVs pumping were observed in response to S-(-)-Bay K8644, an agonist of L-type calcium channels, in a lymphedema model prepared by surgical ligation of a single vessel (Fig. 4c, d). Nano-fluorescence is a platform for investigating the physiology and function of LVs, providing an approach for exploring the discovery of drugs to treat lymphatic dysfunction.

**Fig. 5:**
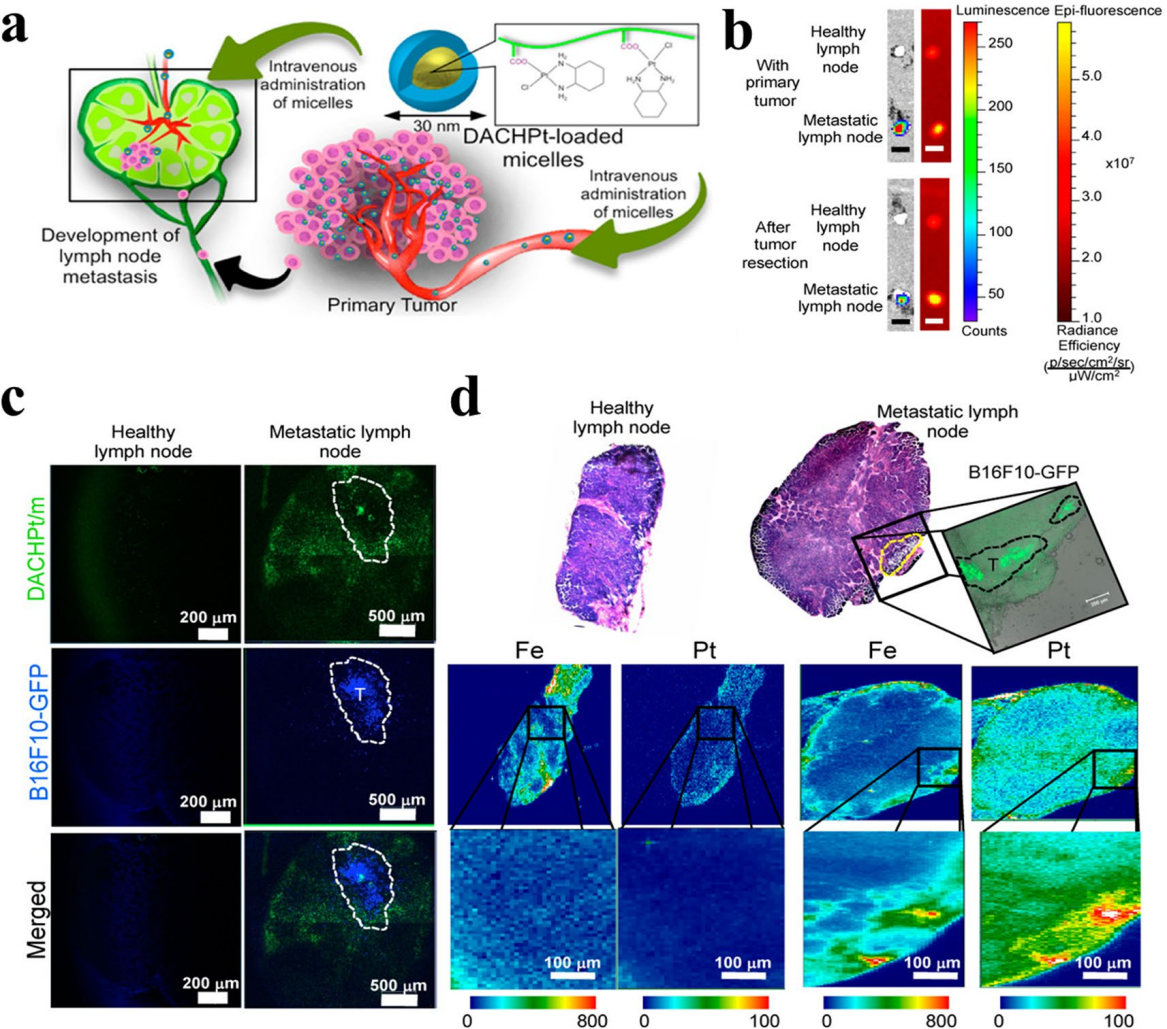
Investigation of DACHPt-loaded micelles (DACHPt/m) in metastatic LNs. (**a**) A schematic pathway of DACHPt/m in lymphatic delivery. (**b**) Ex vivo luminescence/fluorescence images comparing healthy and metastatic LNs after administration of Alexa 647-labeled DACHPt/m. (**c**) Fluorescent microscopy images of healthy and metastatic LNs 24 h after injection of Alexa 647-labeled DACHPt/m (green). B16-F10-GFP cells are indicated in blue (dotted line). (**d**) Micro-distribution of Pt and Fe assessed by µ-synchroton radiation-X-ray fluorescence (µ-SR-XRF) in healthy and metastatic LNs 24 h post-injection of DACHPt/m at a dose of 20 mg/kg. Fluorescence microscopy revealed the presence of B16-F10-GFP metastasis (green; dotted line). (This figure is reproduced from [122], an open-access article distributed under the terms of the Creative Commons Attribution [CC BY-NC] license.)

**Fig. 6:**
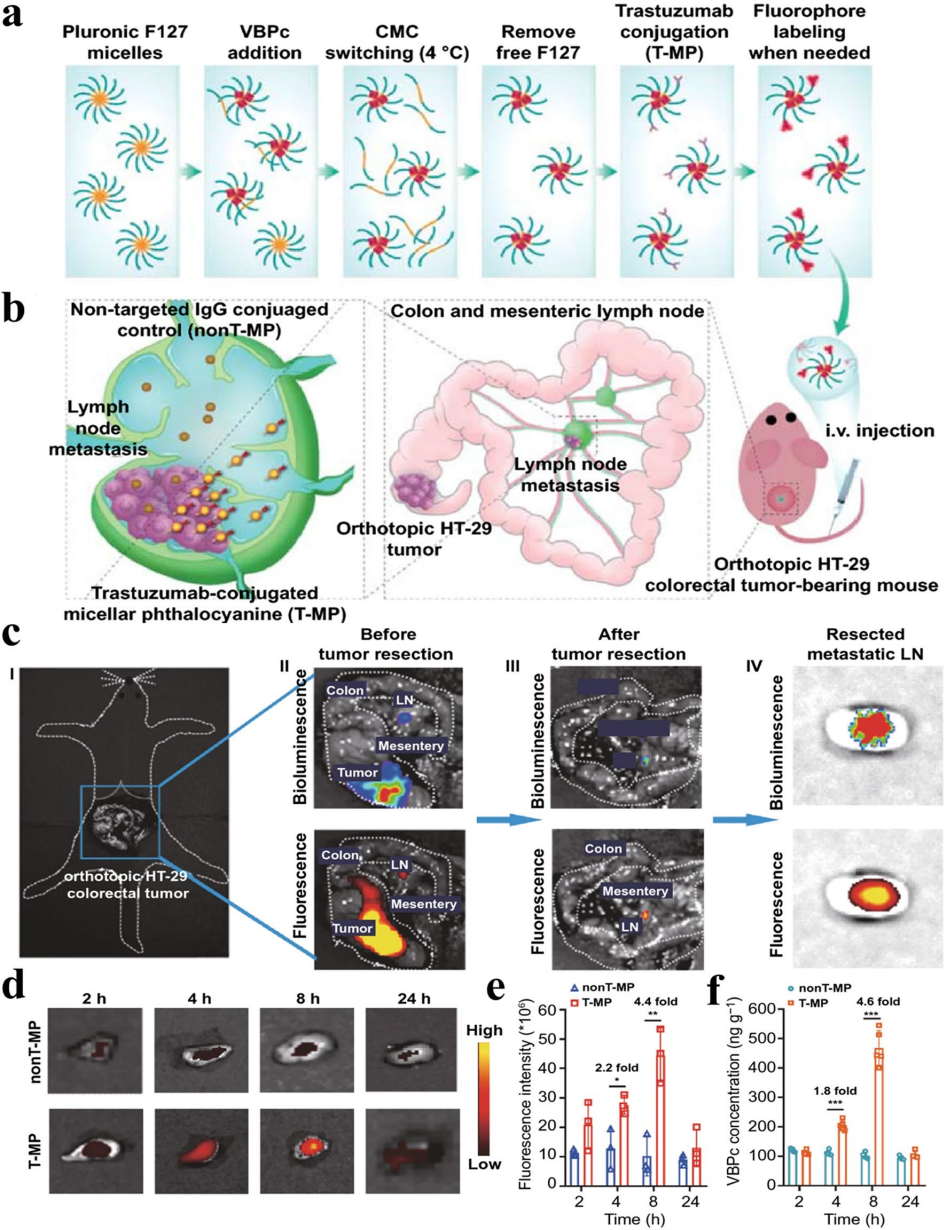
Fluorescence imaging of metastatic LN with Cy5.5-T-MP in orthotopic HT-29-luc colorectal cancer model. (**a**) Schematic preparation of Cy5.5-T-MP. (**b**) Schematic strategies of Cy5.5-T-MP targeting metastatic LNs. (**c**) Targeting and visualization of the metastatic, mesenteric SLN using Cy5.5-T-MP after intravenous administration. (**d**) Ex vivo imaging of Cy5.5-T-MP within the metastatic SLNs in 2,4,8,24 h. (**e**) Quantification of fluorescence intensity of ex vivo SLNs. (**f**) Quantification of photothermal and photoacoustic agent delivered by Cy5.5-T-MP into SLNs. This figure is reproduced from [124], an open-access article distributed under the terms of the Creative Commons Attribution (CC BY-NC) license

Nano-fluorescence that extravasates from blood vessels and exhibits passive transport to LNs can be more efficiently accumulated in LN-metastasized tumors owing to the EPR effects [121]. Indeed, ex vivo fluorescence imaging of (1,2-diaminocyclohexane)platinum(II) (DACHPt)-loaded micelles (DACHPt/m) with a 30-nm diameter showed significantly higher accumulation in metastatic LNs than in healthy LNs, resulting in a superior anti-tumor effect against LN metastasis (Fig. 5a) [122, 123]. In detail, significantly higher accumulation of DACHPt/m in metastatic LNs compared to healthy LNs was observed through ex vivo luminescence and fluorescence imaging after the administration of Alexa 647-labeled DACHPt/m. Moreover, in the animal model bearing both primary tumors and metastatic LNs, consistent selective accumulation of DACHPt/m in the metastatic LNs was observed, regardless of the presence of the primary tumor (Fig. 5b). Examination of the microdistribution of the micelles after intravenous administration revealed no accumulation in healthy LNs, while micelles were found to be distributed throughout the entire section of metastatic LNs (Fig. 5c). Additionally, the overlap of platinum (Pt) from the micelles with iron (Fe) from blood heme proteins in the LN metastasis suggests that the micelles may reach the metastasis via the bloodstream (Fig. 5d). Surface modifications with ligands specific to biomolecules and cells also contribute to the enhanced imaging of LN metastasis. For example, an NIR dye (Cy5.5)-labeled trastuzumab-conjugated micellar phthalocyanine (T-MP) was developed (< 50 nm) by conjugating trastuzumab to HER2-overexpressing cancer cells (Fig. 6a, b) [124]. A strategy was devised to enhance metastasis-specific nanoparticle accumulation in the orthotopic HT-29-luc colorectal model, which predominantly accompanies mesenteric LN (MLN) metastasis, by using small nanoparticles with HER2 ligands targeting the receptors overexpressed on the tumor cells. As a result, successful targeting of the metastatic mesenteric SLNs by T-MP was confirmed through bioluminescence/fluorescence imaging (Fig. 6c). Moreover, Cy5.5-labeled T-MP (Cy5.5-T-MP) exhibited a significantly higher distribution in metastatic SLNs than non-targeted micelles (Cy5.5-nonT-MP) over time (with a peak at 8 h) (Fig. 6d-f). Thus, therapeutic drugs in micelles can be efficiently delivered to metastatic SLNs. Prior studies highlight the potential applications of nano-fluorescence for tracking therapeutic drug delivery into LNs.

Several studies have utilized nano-fluorescence to investigate the mechanisms of drug uptake into immune cells within LNs [52, 125–127]. Tyrosinase-related protein 2 (Trp2)-loaded polyethyleneimine 2k-stearic acid (PSA) micelles with a size smaller than 50 nm were designed to load lipophilic fluorescent dye 1,1’-dioctadecyl-3,3,3’,3’-tetramethylindodicarbocyanine (DiD), with an excitation peak at 646 nm and emission peak at 663 nm [125, 126]. DiD-labeled PSA micelles specifically accumulate in draining LNs (dLNs). Fluorescein isothiocyanate (FITC) was further loaded into DiD-labeled PSA micelles as a model drug to track the uptake pathway of Trp2. FITC fluorescence was concentrated not only in the medulla, where the afferent and efferent LVs are located, but also in the paracortex, where T cells reside. These results explain the efficient T-cell-mediated immune response induced by Trp2-loaded PSA micelles, highlighting their potential in melanoma immunotherapy. In another example involving mannose-modified steric acid-grafted chitosan (MChSA) micelles with a sub-100 nm diameter [127], in vivo fluorescence imaging showed that Cy5-labeled MChSA micelles highly overlapped with CD11c antibody-labeled DCs in TdLNs, indicating the enhanced cellular uptake of the micelles by DCs in TdLNs and the efficient T cell response. Further studies using nano-fluorescence would facilitate the demonstration of the pathway and fates of drugs in LNs, which would accelerate the development of diagnostic agents and LN-based cancer immunotherapeutic drugs.

#### Liposomes

Liposomes are spherical lipid vesicles consisting of phospholipid bilayers that form a hollow structure in aqueous solutions [84, 96, 99]. Because of their biocompatibility, controllable size, modifiability, and biodegradability, liposomes have been extensively studied as drug delivery carriers capable of encapsulating various substances within their hollow cores and lipid bilayers (Fig. 3). The FDA has approved numerous liposomal formulations—such as Ambelcet^®^ (in 1995), Doxil^®^ (in 1995), DaunoSome^®^ (in 1996), Ambisome^®^ (in 1997), Amphotec^®^ (in 1997), DepoCyt^®^ (in 1999), Caelyx^®^ (in 1999), Visudyne^®^ (in 2000), DepoDur^®^ (in 2004), Marqibo^®^ (in 2012), LipoDox^®^ (in 2013), Onivyde^®^ (in 2014), CYXEOS^®^ (in 2017), Shingrix^®^ (in 2017), Arykayce^®^ (in 2018), ONPATTRO^®^ (in 2018), COMIRNATY™ (in 2021), and SPIKEVAX™ (in 2022)—to treat various diseases, underscoring the clinical relevance of liposomes [42, 128–130].

Liposomes of suitable size (< 150 nm) are among the platforms most widely used to develop imaging agents to target the lymphatic system following subcutaneous administration. Similar to other nanomaterials, different targeting strategies (e.g., size optimization, targeting ligands) are often employed in conjunction with liposomes to enhance the specificity and effectiveness of lymphatic imaging (Table 1). For instance, LyP-1 (CGNKRTRGC), a cyclic peptide that exhibits specific binding affinity to the p32 receptor overexpressed on tumor cells and lymphatics, was conjugated to liposomes (L-LS), followed by the loading of DiR (L-LS/DiR) [131]. NIR in vivo immunofluorescence imaging revealed that liposomes approximately 90 nm in size significantly increased dye accumulation in metastatic LNs compared to control liposomes without ligands (LS).

**Fig. 7:**
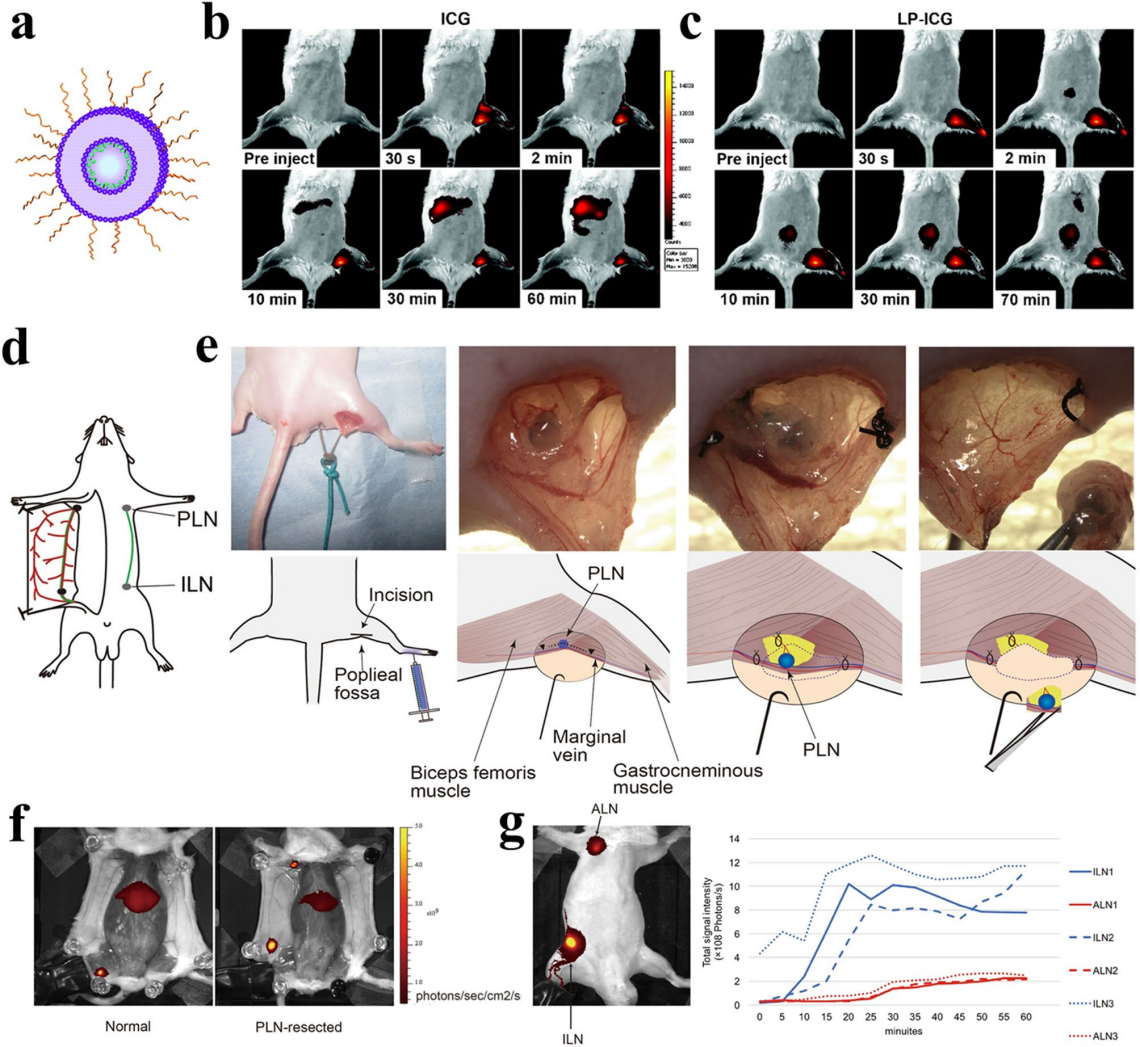
In vivo lymphatic fluorescence imaging using liposomal ICG (LP-ICG). (**a**) A schematic illustration of LP-ICG. (**b**, **c**) Dynamics of ICG signal after intradermal injection in normal C57BL/6 albino mice. Time series of images from representative mice are shown before and after the intradermal injection of ICG and LP-ICG in the left rear paw. (**d**) A schematic diagram defining the ILN in relation to the proper ALN. (**e**) Representative intravital images and schemes displaying the resection procedure for the PLN. After injecting Evans blue dye into the left footpad as a guidance dye, a 10-mm incision was made at the popliteal fossa. A black arrowhead indicates the LVs adjacent to the marginal vein. Ultimately, the PLN, along with its connecting blood and LVs, was excised. (**f**) Representative images showing the distribution of DiR-labeled liposomes in mice with PLN resection compared to the control mice. (**g**) Time-dependent changes in fluorescent signals within the ILN and ALN. This figure was reproduced from [135] for (**a-c**), and [139] for (**d-g**), open-access articles distributed under the terms of the Creative Commons Attribution (CC BY-NC) license

Researchers have also utilized ICG to develop in vivo SLN imaging liposomal tracers owing to its excellent biosafety (as verified by the FDA) and ease of formulation with serum proteins [132–138]. However, ICG is a dim dye because of its tendency to aggregate and self-quench in solution. In addition, ICG may inhibit lymphatic contraction. To address these limitations, liposomal ICG (LP-ICG) (58.8 ± 1.8 nm) was proposed by formulating ICG with DSPE-PEG and 2-dioleoyl-*sn*-glycero-3-phosphocholine (DOPC) (Fig. 7a) [135, 136]. These liposome formulations provided greater stability to ICG in solution and biological fluids, resulting in enhanced fluorescence signals with a shift towards longer wavelengths for both absorption and emission compared to free ICG. In vivo NIR fluorescence imaging demonstrated that the intradermal injection of LP-ICG into C57BL/6J-*Tyr*^*c−J*^ albino mice facilitated visualization of the second dLN, which was located significantly deeper (approximately 1 cm) than the PLN; however, this was not observed with free ICG (Fig. 7b, c) [135]. Owing to its more specific uptake by the lymphatic system than free ICG, LP-ICG also enabled the visualization of lymphatic flow and clearance. In another study by the same group, NIR fluorescence imaging of both popliteal collecting LVs and PLN was performed after administering LP-ICG to a B16 footpad tumor-bearing mouse with LN metastasis [136]. Imaging indicated that the fluorescence signal intensity in the LN increased over time. Specifically, LP-ICG perfused the pre-collector LVs but did not drain into the larger collecting vessels. These results suggest that LP-ICG flow through the LN is impeded, implying that lymphatic flow in the primary dLN can be obstructed as metastases develop. This obstruction may cause lymph flow to reroute to alternative LNs, which could be associated with the spread of cancer cells to other sites and low response rates to immunotherapeutic agents.

Nano-fluorescence can also be used to establish LN-to-LN trafficking. DiR-labeled liposomes with a particle size of 114 ± 5 nm were administered to the footpads of mice; PLNs and their efferent and afferent LVs, marginal veins, and feeding blood vessels were resected (Fig. 7d, e) [139]. Despite the absence of popliteal lymphatic systems, DiR-labeled liposomes were quantitatively detected in axillary LN (ALN) by detouring through the inguinal LN (ILN) (Fig. 7f, g).

In sum, liposomal nanoformulations not only overcome the issues of self-quenching and potential inhibition of lymphatic function but also facilitate lymphatic imaging. In particular, prior studies provide mechanistic insights into the lymphatic physiology and dysfunction that govern disease prognosis and treatment response rates.

#### Polymersomes

Polymersomes are self-assembled amphiphilic vesicles similar to liposomes but are composed of block copolymers with hydrophobic and hydrophilic segments instead of phospholipids [140–142]. Polymersomes serve as useful delivery platforms for various drugs and imaging agents due to their high stability and capacity, as described in Fig. 3, for hydrophobic and hydrophilic substances (Table 1). A study investigating the pharmacokinetics of QD-labeled, size-controlled, and long-circulating polymersomes in dLNs demonstrated promising results [143]. Cholinic acid or saturated fatty acids have been conjugated with chitosan to prepare water-soluble polymersomes, which were subsequently labeled with QDs. The fluorescence intensity of QD705-labeled polymersomes exhibited a half-life of 6 ± 2 h in the bloodstream and 11 ± 3 h in the LNs. Peak fluorescence intensity in the LNs was observed within the first hour post-injection, plateaued over the next 3 h, and then gradually declined over the subsequent 3–24 h. These data suggest that size-controlled, long-circulating polymersomes have significant potential as imaging-agent carriers for LN mapping, leveraging their enhanced chemical stability and versatility. An important study exploiting the high stability of polymersomes involved tracking mature DCs in LNs [144]. NIR-emissive polymersomes were designed by segregating NIR fluorophore into the hydrophobic bilayer core composed of poly(ethylene oxide(1300)-*b*-butadiene(2500)) block copolymers incorporating 4-fluoro-3-nitrobenzoic acid. The Tat peptide was further modified onto polymersomes to enhance the delivery of NPs to DCs. Mature murine DCs were labeled with Tat-NIR polymersomes, followed by intravenous and subcutaneous injections. Fluorescence lifetime imaging was used to eliminate autofluorescence, enabling effective tracking of DCs in naïve mice. The right PLN tested positive for NIR-DCs, indicating that DCs remained in the dLNs for up to 33 days. These findings imply that the DCs responsible for antigen uptake and processing can reside in TdLNs long enough to instruct T cells, underscoring the importance of adjuvant techniques along with antigen delivery systems for efficient vaccination and cancer immunotherapy.

In sum, fluorescent polymersomes, with their advantageous properties such as high stability, chemical versatility, and the ability to encapsulate both hydrophilic and hydrophobic drugs, are expected to be further utilized not only to develop efficient therapeutics and imaging techniques but also in mechanistic studies essential for designing effective treatment regimens.

#### Silica NPs

Silica NPs are representative inorganic NPs that offer distinctive advantages such as biocompatibility, high stability, tunable size, porosity, and notably, versatile silane chemistry that allows for simple functionalization with drugs and imaging agents, as shown in Fig. 3 (Table 1) [96]. Several studies have explored the potential use of fluorescence-doped silica NPs for SLN mapping [30, 145, 146].

**Fig. 8:**
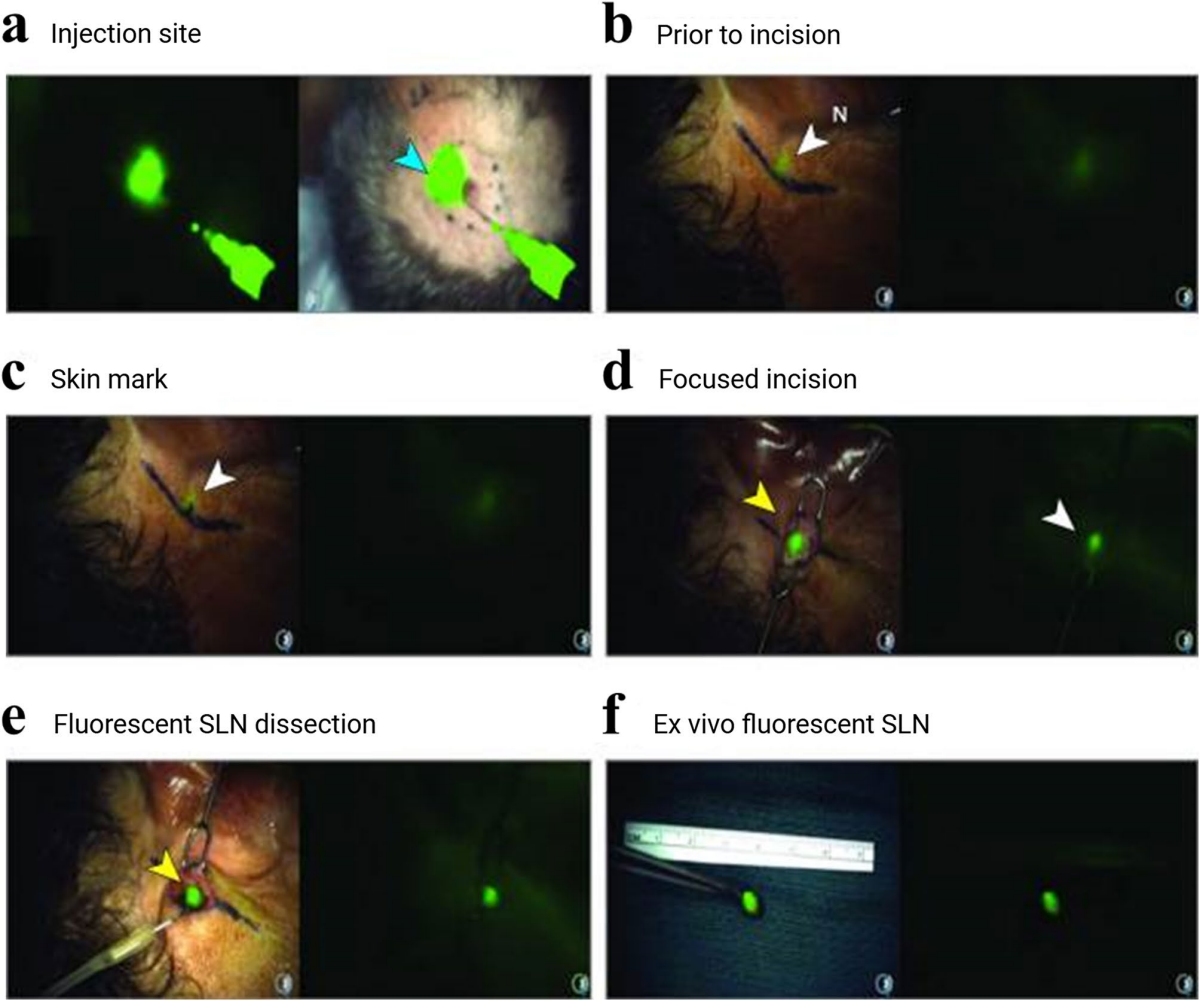
Fluorescent silica NPs for real-time visualization of postauricular SLNs. (**a-f**) Real-time optical imaging using the spectrum camera system (Quest) detected focal fluorescence of the NPs over the postauricular SLN in a male patient in his 50s with scalp melanoma, resulting in limited surgical dissection and a significantly smaller resection cavity than initially planned. This figure was reproduced from [30], an open-access article distributed under the terms of the Creative Commons Attribution (CC BY-NC) license

Silica NPs, known for their high biocompatibility, and tunable size and porosity, were successfully prepared using the Stöber method and doped with Rhodamine B isothiocyanate (RITC) (RITC-SiO2), with a size adjusted to 75 ± 7 nm to prevent the rapid transit of dye through the small size of SLNs and its subsequent spread to more distant LNs [147]. The high stability and colloidal stability of these NPs make them ideal for lymphatic system imaging. After injection into the right footpad of mice, fluorescent signals from the NPs were detected in the ALNs and brachial LNs (BLNs) near the footpad when the skin was removed, whereas no significant signals were observed in other organs. NIR-emitting Cy7-dopped silica NPs with PEG shells have also been developed for in vivo LN imaging [148]. The NPs exhibited high stability in serum, a negative ζ-potential, and a diameter within the 20–80 nm range. Subcutaneous injection of Cy7-dopped silica NPs with a PEG shell into the right anterior paw resulted in high fluorescence signals in the right ALNs (RALNs). Core-shell silica NPs (~ 6 nm) that surfaced were functionalized with Cy5.5; moreover, integrin-targeting peptides—specifically cyclic arginine-glycine-aspartic acid-tyrosine (cRGD), PEGs—were explored as an imaging agent to map SLNs for image-guided surgery in clinical trials (Fig. 8a) [30]. Focal fluorescence was observed through the intact skin over the SLN for real-time optical imaging (Fig. 8b-f). Using fluorescence signaling, the SLN was successfully dissected through an incision site that was more than three times smaller than the initially marked area (Fig. 8c, d). To confirm the feasibility and accuracy of Cy5.5-labeled core-shell silica NPs in image-guided surgery for SLNs, lymphoscintigraphy with technetium Tc^99m^ sulfur colloid was used as a control. Remarkably, the concordance rate between lymphoscintigraphy and image-guided surgery using Cy5.5-labeled core-shell silica NPs was 90% in 40 SLN excisions during the 24 surgical procedures. Recently, in non-human primate models, naked-eye visualized, fluorescence-guided surgical operations using silica NPs in the long term were investigated [149]. Green-emitting fluorescent SiNPs with a size of approximately 5 nm were used, and under UV irradiation, SiNP-stained LVs and ALNs were easily visible to the naked eye. These results highlight the potential of using nano-fluorescence in image-guided surgery to identify and dissect metastatic LNs.

The monoclonal antibody MECA79 specifically binds to peripheral node addressin (PNAd), a sulfated sialyl-LewisX (sLeX)-like sugar expressed on HEVs, and can be exploited for the efficient LN delivery of drugs and imaging agents, even via systemic administration. Accordingly, DiR-labeled tacrolimus (FK506) drug-loaded mesoporous silica (MSN) NPs with MECA79 (DiR-MSN-FK506-MECA79) were prepared [150]. Ex vivo fluorescence imaging of MLNs, ILNs, and ALNs 24 h after tail vein injection showed that the MECA79-modified NPs exhibited significantly higher fluorescence intensity in all LNs than NPs without MECA79. This study proposes a method to develop nano-fluorescence to detect various LNs located throughout the body following systemic administration.

Silica-coated iron oxide (SCION) NPs with fluorescent dyes (Cy5.5 and AlexaFluor555), ranging in size from 18 to 20 nm, were developed as dual-imaging modalities [145]. After subcutaneous injection, the NPs mostly migrated passively to the LNs, facilitating enhanced visibility in the optical imaging of the draining SLNs due to their ability to perform both fluorescence imaging and MRI. The nano-fluorescence-based dual-imaging modality can provide an opportunity to confirm the accuracy of fluorescence imaging by comparing it with other imaging modalities.

**Table 1:** Fluorescent dye-incorporating NPs

Formulation	Fluorescence dye	NP components	Disease model (potential diseases)	Applications	Ref.
Dendrimer	Cy5.5 dye	PAMAM dendrimer with Gd-(III)-DTPA chelates	-	Image-guided surgery of SLN; MRI and fluorescence imaging in dual modality	[98]
Polymeric micelle	DOX, DiR	DSPE-PEG micelle	LN metastasis	In vivo imaging of micelle accumulation within PLNs	[110]
Polymeric micelle	IR780	DSPE-PEG micelle modified with mannose	Breast cancer, LN metastasis	In vivo NIR fluorescence imaging for targeting TAM surface CD206 in metastatic LNs	[111]
Polymeric micelle	AlexaFluor 488	Pluronic-PPS	Solid tumors, LN metastasis	Fluorescence microscopy of lymphatic draining Pluronic-PPS NPs accumulation in the TdLN	[112]
Polymeric micelle	AlexaFluor 647	S-nitrosated PPS	(Lymphatic-related cancer, infectious disease)	Fluorescence imaging in draining LN with SNO-NP	[113]
Polymeric micelle	IRDye 680	PPS/PPG-PEG micelle	Lymphedema	Fluorescence imaging of LVs in lymphedema model	[114]
Polymeric micelle	IRDye 680RD	S-nitrosated PPS	Lymphedema	Fluorescence imaging to detect transport and NP accumulation within dLNs	[115]
Polymeric micelle	AlexaFluor 647, Dir	DACHPt-loaded PEG-*b*-P(Glu) micelle	Melanoma, LN metastasis	Ex vivo fluorescence imaging of DACHPt/m accumulation in metastatic LNs and healthy LNs	[122]
Polymeric micelle	AlexaFluor 680	DACHPt-loaded PEG-*b*-P(Glu) micelle	Grastric cancer, LN metastasis	Whole-body NIR fluorescence imaging and ex vivo metastatic LNs imaging with DACHPt/m	[123]
Polymeric micelle	Cy5.5	T-MP	Colorectal tumor, LN metastasis	Ex vivo fluorescence imaging of Cy5.5-T-MP accumulation in metastatic SLNs	[124]
Polymeric micelle	DiD	Trp2/CpG-loaded hybrid micelle	Lung metastatic melanoma	Fluorescence imaging with micelle targeting dLN	[125]
Polymeric micelle	DiD, FITC	Trp2-loaded PSA micelle	Melanoma	In vivo fluorescence microscopy for evaluating micelle accumulation in dLN and tracking the uptake pathway	[126]
Polymeric micelle	Cy5	MChSA micelle	Melanoma	In vivo fluorescence imaging for quantifying cellular uptake of micelle by DC in TdLN	[127]
Polymeric micelle	CdTeSe/CdZnS QD	PEG/PEG-COOH micelle	(Breast cancer)	In vivo NIR fluorescence imaging of QD accumulation in ALNs and TLNs	[151]
Liposome	DiR	LyP-1-PEG-DSPE liposome	LN metastasis	In vivo NIR fluorescence imaging of L-LS/DiR distribution in metastatic PLNs	[131]
Liposome	ICG	DOPC/PEG-DSPE liposome	Melanoma	In vivo NIR fluorescence imaging of the LP-ICG in PLN, ILN, and second dLN	[135]
Liposome	ICG	DOPC/PEG-DSPE liposome	Melanoma, LN metastasis (breast cancer)	In vivo NIR fluorescence imaging to visualize popliteal-collecting LVs and PLN	[136]
Liposome	DiR	EPC: Chol: DMG-PEG	Lymphedema	In vivo fluorescence for LN-to-LN trafficking	[139]
Polymersome	QD705	Cholanic acid-modified chitosan polymersome	Colorectal cancer	In vivo fluorescence imaging of long circulating QD-labeled polymersomes in dLNs	[143]
Polymersome	4-fluoro-3-nitrobenzoic acid	Tat-modified poly(ethylene oxide (1300)-*b*-butadiene(2500))	-	NIR fluorescence imaging for DC-tracking in dLNs	[144]
Silica NP	Cy5.5	cRGDY-PEG NP	Head and neck melanoma	Fluorescence-guided SLN biopsy in head and neck melanoma patients	[30]
Silica NP	Cy5.5, AlexaFluor555	SCION	-	In vivo draining SLN imaging with SCION; dual-imaging modality MRI and optical imaging	[145]
Silica NP	ICG	PAMAM-coated silica NP	-	LN dissection in the neck; radiography and fluorescence imaging in dual modality	[146]
Silica NP	RITC	^68^Ga-NOTA-SiO_2_	(LN metastasis)	In vivo fluorescence imaging of SLN with RITC-SiO_2_ NPs and ex vivo imaging of ALNs and BLNs	[147]
Silica NP	Cy7	Pluronic-based core–shell silica-PEG NP	-	In vivo NIR fluorescence imaging in RALNs	[148]
Silica NP	DiR	MSN-FK506-MECA79	Transplantation	Ex vivo fluorescence imaging of MLNs, ILNs, and ALNs for treating LN-targeted transplant rejection	[150]

### Self-luminescence/fluorescence NPs

Various studies using fluorescent dye-incorporated NPs have shown great potential for clinical applications. However, they still face limitations that must be addressed for practical use, such as their inability to eliminate autofluorescence and the propensity of some fluorescent dyes to undergo self-quenching. Accordingly, self-luminescent and self-fluorescent NPs have emerged as alternatives to fluorescent dye-incorporating NPs. In this section, we discuss LN imaging with self-fluorescent NPs (e.g., QDs and UCNPs).

#### QDs

QDs are semiconductor particles with dimensions typically ranging from 2 to 10 nm and are composed of noble metals such as cadmium selenide (CdSe), cadmium telluride (CdTe), and indium arsenide (InAs) [84, 152–154]. QDs not only exhibit high fluorescence with a high quantum yield but also display varying fluorescent wavelengths depending on their size and composition. In contrast to general fluorescent dyes, QDs offer an intense, stable, and long-lasting fluorescent signal at a low cost and are less prone to self-quenching (Fig. 3) [153, 155, 156]. These physicochemical properties make them especially suitable for In vivo optical imaging and diagnostic applications [157, 158]. Indeed, various QDs with peak emission wavelengths of 565 nm (Qdot 565 ITK), 605 nm (Qdot 605 ITK), 655 nm (Qdot 655 ITK), 705 nm (Qdot 705 ITK), and 800 nm (Qdot 800 ITK) have been demonstrated to visualize various LNs, including ALNs, thoracic LNs (TLNs), deep cervical LNs, superficial cervical LNs, and lateral thoracic LNs (Table 2).

A notable study employing QDs for two-color spectral fluorescence lymphatic imaging utilized QD^®^705 and QD^®^800 [159]. In this study, LVs were visualized, and lymphatic drainage territories were delineated by observing the ALNs, lateral TLNs, and cervical LNs (CLNs) after QD^®^705 was injected into the breast and QD^®^800 was administered to the upper extremity. Consequently, two of the ALNs displayed fluorescence exclusively from QD^®^800, suggesting that lymphatic flow in these nodes originated solely in the upper extremity. Conversely, when QD^®^800 was injected into the breast and QD^®^705 into the upper extremity, lymphatic drainage from the breast was only faintly visualized, with QD^®^705 being distinctly observed. This outcome supports the hypothesis that QD^®^705, with its smaller core diameter of approximately 6 nm and a thin coating of 1–2 nm, is well-suited for imaging lymphatic drainage from the breast, while QD^®^800, with a larger core diameter of about 12 nm, is more appropriate for visualizing lymphatic drainage from the upper extremity. These observations highlight the effectiveness of matching QD sizes to specific lymphatic basins for enhanced imaging accuracy and underscore the importance of selecting appropriate nano-fluorescence sizes to optimize the visualization of lymphatic flow in different anatomical regions.

The NIR spectral window can be categorized into NIR-I (700–1000 nm) and NIR-II (1000–1700 nm) [57, 160]. NIR-II light exhibits less scattering, absorption, and autofluorescence by biomolecules, cells, and tissues, allowing for deeper penetration than NIR-I light. NIR-II fluorescent QDs were employed for LN mapping, with their initial use documented in *Nature Biotechnology* (2004) [161]. NIR-II QDs were engineered to display fluorescence emission in the range of 840–860 nm with a diameter of 15–20 nm. When NIR-II QDs were intradermally injected into the thighs of pigs, the SLNs located approximately 1 cm beneath the skin’s surface were successfully identified.

**Fig. 9:**
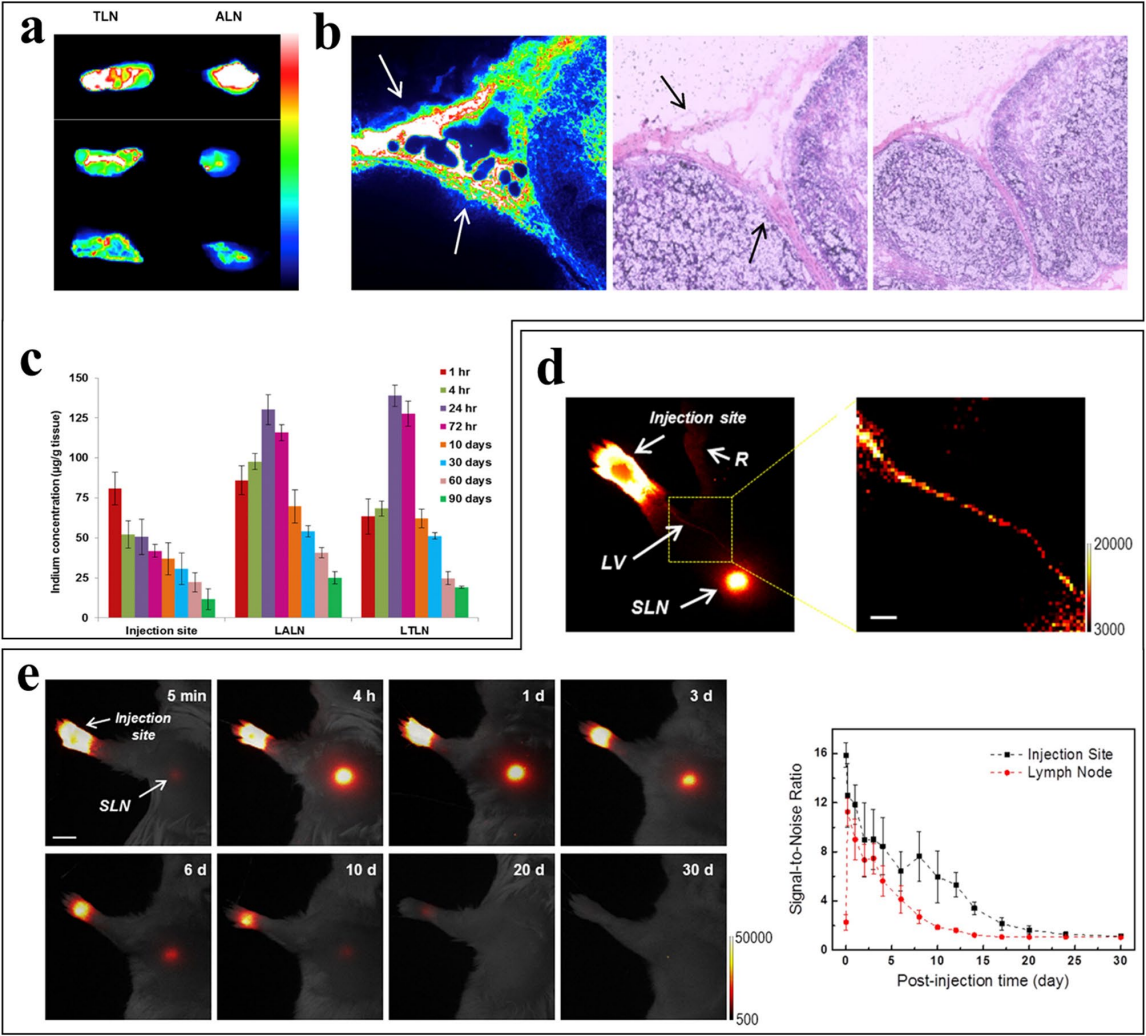
QDs and UCNPs in LN detection. (**a**) Ex vivo images of ALNs and TLNs harvested after subcutaneous QD injection (30 pmol/g) at 4 h, 48 h, and 5 days post-injection, illuminated with a 455-nm LED for 0.1 s. (**b**) A photoluminescence image (left, 250 × 250 μm) of the rat ALN 5 min post-injection of QDs, alongside a corresponding hematosylin and eosin (H&E) staining image (middle) and a lower power image (right). These showed high fluorescence in the SCS (top arrows) and trabecular regions (bottom arrows). (**c**) Quantitative QD biodistribution, assessed via inductively coupled plasma-mass spectrometry (ICP-MS), measuring indium content (the quantity of indium per gram of tissue) in the LALN, LTLN, and injection sites (mean ± SD, *n* = 3 per group). (**d**) An In vivo UCL image post-injection of surface-modified UCNPs, showing the LV and SLN, with reflected secondary emission (R) from the injection site. A magnified UCL image of the LV on the right. Pixel size, ~ 120 × 120 μm; scale bar, 1 mm. (**e**) In vivo UCL images of the LNs over time after UCNP injection into the forepaw footpad, comparing signal-to-noise ratios between the ALN and injection site (*N* = 4); scale bar, 5 mm. This figure is reproduced from [163] for (**a-c**) and [172] for (**d**, **e**), an open-access article distributed under the terms of the Creative Commons Attribution (CC BY-NC) license

A significant concern in the clinical translation of QDs is their potential toxicity, largely due to the inclusion of toxic noble metals in their cores [162]. To address this challenge, indium (In)-based bio CFQD^®^ NPs with an emission range of 500–700 nm were developed, offering biocompatibility while maintaining a high photoluminescence quantum yield [163]. When injected subcutaneously into the left paw of rats, ex vivo fluorescence imaging facilitated the detection of left axillary LN (LALN) and left thoracic LN (LTLN), similar to the observations reported for other types of QDs (Fig. 9a). The QDs were primarily localized in the SCS region, where metastatic carcinoma commonly occurs in LNs (Fig. 9b). Notably, QD-injected rats displayed clear fluorescence signals in both the axillary and thoracic LNs 10 days post-injection (Fig. 9c). These results suggest that bio CFQD^®^ NPs remained relatively intact, sustaining detectable fluorescence signals over a prolonged period and were eventually cleared from the body. In addition to the development of novel types of QDs, several strategies have been proposed to minimize the toxicity of NIR QDs, including increasing the fluence rate while proportionally reducing the NIR QD dosage and minimizing exposure to QD materials by modifying their surfaces [161, 164].

Autofluorescence can diminish the effectiveness of In vivo fluorescence imaging by reducing the contrast between the target and background signals. Although QDs can effectively reduce autofluorescence by producing ultrabright fluorescence, an external light source is still required for excitation, making it challenging to eliminate autofluorescence completely in in vivo fluorescence imaging. Bioluminescence resonance energy transfer (BRET) is emerging as a technique to effectively reduce autofluorescence effects because it does not require an external light source [165]. BRET transfers energy between a light-emitting enzyme and fluorescent molecule. Since its debut in 1999, BRET has evolved with various donor–acceptor pairs and is now widely used to study protein interactions in living cells. This is valuable for screening new drugs and identifying inhibitors of protein-protein interactions, which are important for developing new therapies. Accordingly, BRET QDs have been developed to precisely image lymphatics while mitigating autofluorescence issues. BRET-QD655 emits NIR light, resulting in better tissue penetration and reduced signal loss within the tissue. Unlike conventional fluorescence techniques, BRET-QDs generate minimal autofluorescence, resulting in clearer visualization of all LNs with lower background noise than bare QDs [166]. This technology provides more accurate quantitative imaging than fluorescence imaging by directly reflecting the concentration of QDs without the need for an excitation light. However, further studies are required to address the stability issues of the BRET-QD system.

#### UCNPs

UCNPs, typically doped with lanthanide ions, have recently gained considerable attention owing to their remarkable photostability, deep-tissue penetration, and biocompatibility (Fig. 3) [167, 168]. UCNPs are characterized by their unique ability to emit light at a wavelength shorter than the excitation wavelength through sequential absorption of two or more photons [169]. This distinguishes them from conventional fluorescent materials that exhibit a Stokes shift and generally emit light at wavelengths longer than the excitation wavelength. This up-conversion process enables highly sensitive detection. Additionally, similarly to QDs, UCNPs facilitate the detection of biological tissues located at substantial penetration depths due to their light emission in the NIR region.

Studies have investigated the potential of UCNPs for long-term, non-invasive, In vivo LN optical imaging (Table 2) [170–172]. The movement and retention of Tm-doped UCNPs within the lymphatic system were assessed using high-resolution NIR-to-NIR upconversion luminescence (UCL) imaging after subcutaneous injection into the mouse footpad [172]. The intensity of the fluorescence signals at the LVs, SLNs, and whole body was monitored over time post-injection (Fig. 9d). The UCNPs rapidly migrated to and accumulated in the SLN within 3–4 days, traveled through the lymphatic system to the bloodstream, and were eventually excreted from the body approximately one month later (Fig. 9e). Hence, UCNPs could provide an alternative, non-invasive, and highly sensitive method for long-term optical LN imaging in vivo.

**Table 2:** Self-luminescence/fluorescence NPs

Formulation	Particle type	Potential diseases or techniques	Applications	Ref.
QD	QD^®^705, QD^®^800	Breast cancer, lymphedema	Two-color NIR fluorescence imaging to visualize dLNs	[159]
QD	NIR-II QDs	Cancer	NIR fluorescence imaging for SLN mapping in pig models	[161]
QD	Bio CFQD^®^	Breast cancer	Ex vivo fluorescence imaging of LALN, LTLN	[163]
QD	BRET-QD655	Cancer	In vivo, ex vivo fluorescence imaging of BRET-QD655 in LN and lymphatic duct	[166]
QD	QD655, QD585, QD545, QD565, QD605	Clinical surgery	In vivo / in situ / ex vivo multicolor fluorescence imaging of neck LNs	[173]
UCNP	NaYF4:Yb, Er (Tm) nanocrystal	Cancer	In vivo luminescence lymphatic imaging with UCNP in dLNs	[170]
UCNP	NaYF4:Yb, Er (Tm) nanocrystal	Cancer, LN metastasis	In vivo multicolor UCL imaging for cancer cell tracking and lymph node mapping	[171]
UCNP	NaYF4:Yb, Tm nanocrystal	SLN biopsy	In vivo long-term UCL imaging of SLN and LV with UCNPs	[172]

## Conclusions

Nano-fluorescence imaging is a promising tool for visualizing lymphatic structures and functions, particularly in addressing the limitations posed by traditional imaging modalities. This technology offers distinct advantages such as naked-eye visibility, real-time imaging, non-invasiveness, tunable size, surface functionality, and diverse color spectra. Various nano-fluorescence techniques have demonstrated significant potential for improving diagnostic accuracy in lymphatic-related diseases. Clinical applications, including the use of FDA-approved fluorescent dyes and NP-based imaging agents, underscore the practical utility of nano-fluorescence for diagnosing and treating lymphatic disease. Despite challenges such as photobleaching, limited tissue penetration, and biocompatibility concerns, ongoing innovations in NP technology and imaging techniques are addressing these issues. Additionally, nano-fluorescence has expanded our understanding of lymphatic physiology and immune cell uptake mechanisms within LNs and the connectivity between LNs, creating new opportunities to leverage them to develop novel immunotherapeutic approaches for controlling lymphatic-related diseases. These innovations not only enable dual diagnostic and therapeutic functions but are also expected to play a significant role in personalized medicine and early diagnosis and treatment strategies. With continued research addressing biosafety, effectiveness in lymphatic drainage, specificity to target organ and cells, and signal-to-noise, the development of clinical nano-fluorescence platforms could soon extend beyond cancer to encompass the diagnosis and treatment of various lymphatic-associated diseases. Such progress holds great promise for improving diagnostic and therapeutic efficacy, enhancing patient compliance, and ensuring safety in clinical settings. Moving forward, the integration of nano-fluorescence into standard diagnostic protocols has the potential to revolutionize the field of lymphatic imaging, enhancing both therapeutic outcomes and patient care.

## Electronic supplementary material

Below is the link to the electronic supplementary material.

Supplementary Material 1

## Abbreviations

LN: Lymph node
LV: Lymphatic vessel
TdLN: Tumor draining lymph node
SLN: Sentinel lymph node
US: Ultrasound
MRI: Magnetic resonance imaging
CT: Computed tomography
SPECT: Single photon emission computed tomography
PET: Positron emission tomography
QD: Quantum dot
NIR: Near-infrared
UCNP: Upconversion nanoparticle
NP: Nanoparticle
EPR: Enhanced permeability and retention
PEG: Poly(ethylene glycol)
ICG: Indocyanine green
5-ALA: 5-aminolevulinic acid
FDA: Food and Drug Administration
LEC: Lymphatic endothelial cell
ECM: Extracellular matrix
SCS: Subcapsular sinus
HEV: High endothelial venule
FRC: Fibroblast reticular cell
NK: Natural killer
DC: Dendritic cell
PAMAM: Polyamidoamine
PPI: Poly(propylenemine)
Gd: Gadolinium
DSPE-PEG: 1,2-distearoyl-sn-glycero-3-phosphoethanolamine-poly(ethylene glycol)
DOX: Doxorubicin
MPEG-DSPE/DOX: Doxorubicin loaded 1,2-distearoyl-sn-glycero-3-phosphoethanolamine-poly(ethylene glycol) micelle
DiR: 1,1’-dioctadecyl-3,3,3’,3’-tetramethyl indotricarbocyanine iodide
PLN: Popliteal lymph node
TAM: Tumor associated macrophage
MR780: Mannose-IR780 conjugated
PPS: Poly(propylene sulfide)
PPG: Poly(propylene glycol)
DACHPt: (1,2-diaminocyclohexane)platinum(II)
DACHPt/m: (1,2-diaminocyclohexane)platinum(II)-loaded micelle
T-MP: Trastuzumab-conjugated micellar phthalocyanine
Cy5.5-T-MP: Cy5.5-labeled trastuzumab-conjugated micellar phthalocyanine
Trp2: Tyrosinase-related protein 2
PSA: Polyethyleneimine 2k-stearic acid
DiD: 1,1’-dioctadecyl-3,3,3’,3’-tetramethylindodicarbocyanine
dLN: Draining lymph node
FITC: Fluorescein isothiocyanate
MChSA: Mannose-modified steric acid-grafted chitosan
µ-SR-XRF: µ-synchroton radiation-X-ray fluorescence
L-LS: LyP-1-conjugated liposome
L-LS/DiR: LyP-1-conjugated liposomes/1,1’-dioctadecyl-3,3,3’,3’-tetramethyl indotricarbocyanine iodide
LP-ICG: Liposomal indocyanine green
DOPC: 2-dioleoyl-sn-glycero-3-phosphocholine
ALN: Axillary lymph node
ILN: Inguinal lymph node
RITC: Rhodamine B isothiocyanate
RITC-SiO_2_: Silica nanoparticles doped with Rhodamine B isothiocyanate
BLN: Brachial lymph node
RALN: Right axillary lymph node
cRGD: Cyclic arginine-glycine-aspartic acid-tyrosine
PNAd: Peripheral node addressin
sLeX: Sulfated sialyl LewisX
MSN: Mesoporous silica nanoparticle
MLN: Mesenteric lymph node
SCION: Silica-coated iron oxide nanoparticle
EPC: Egg phosphatidylcholine
CdSe: Cadmium selenide
CdTe: Cadmium telluride
InAs: Indium arsenide
TLN: Thoracic lymph node
CLN: Cervical lymph node
In: Indium
LALN: Left axillary lymph node
LTLN: Left thoracic lymph node
BRET: Bioluminescence resonance energy transfer
UCL: Upconversion luminescence imaging
ICP-MS: Inductively coupled plasma-mass spectrometry
