# Supplementary material for: Nano-fluorescence imaging: advancing lymphatic disease diagnosis and monitoring
Source: Nano Converg. 2024 Dec 11;11:53. doi: 10.1186/s40580-024-00462-1 (PMC11635084; doi:10.1186/s40580-024-00462-1)
Supplement: Supplementary file 1 — Supplementary Material 1 [file 40580_2024_462_MOESM1_ESM.docx]

**Dr. William Jo**

**Editor-in-Cheif**
*Nano Convergence*

Nov 18, 2024

Dear Dr. Jo,

Thank you very much for reviewing our manuscript (Ms. ID: NCON-D-24-00192) entitled, “Nano-Fluorescence Imaging: Advancing Lymphatic Disease Diagnosis and Monitoring”, by Chae Yeon Han, Sang-Hun Choi, Soo-Hyang Chi, Ji Hyun Hong, Young-Eun Cho, and myself for publication on *Nano Convergence*. I appreciate your kind consideration of our manuscript. Also, I thank reviewers very much for the constructive and valuable comments, which have certainly improved the quality of the revised manuscript. We have revised by addressing all the issues raised by the reviewers and responded to reviewer’s comments. Changes have been marked in red in the revised manuscript.

We believe the revised manuscript has been substantially improved to meet your standards and hope it will be accepted for publication in *Nano Convergence*.

With best regards and looking forward to hearing from you soon.

Sincerely,

Jihoon Kim, Ph.D.

Assistant Professor

School of Integrative Engineering

[jihoonkim@cau.ac.kr](mailto:jihoonkim@cau.ac.kr)
